# Hunting for the LCT-13910*T Allele between the Middle Neolithic and the Middle Ages Suggests Its Absence in Dairying LBK People Entering the Kuyavia Region in the 8th Millennium BP

**DOI:** 10.1371/journal.pone.0122384

**Published:** 2015-04-08

**Authors:** Henryk W. Witas, Tomasz Płoszaj, Krystyna Jędrychowska-Dańska, Piotr J. Witas, Alicja Masłowska, Blandyna Jerszyńska, Tomasz Kozłowski, Grzegorz Osipowicz

**Affiliations:** 1 Department of Molecular Biology, Medical University of Łódź, Łódź, Poland; 2 Institute of Physics, Nicolaus Copernicus University, Toruń, Poland; 3 Department of Anthropology, Adam Mickiewicz University, Poznań, Poland; 4 Department of Anthropology, Nicolaus Copernicus University, Toruń, Poland; 5 Department of Archaeology, Nicolaus Copernicus University, Toruń, Poland; University of Iceland, ICELAND

## Abstract

Populations from two medieval sites in Central Poland, Stary Brześć Kujawski-4 (SBK-4) and Gruczno, represented high level of lactase persistence (LP) as followed by the LCT-13910*T allele’s presence (0.86 and 0.82, respectively). It was twice as high as in contemporaneous Cedynia (0.4) and Śródka (0.43), both located outside the region, higher than in modern inhabitants of Poland (0.51) and almost as high as in modern Swedish population (0.9). In an attempt to explain the observed differences its frequency changes in time were followed between the Middle Neolithic and the Late Middle Ages in successive dairying populations on a relatively small area (radius ∼60km) containing the two sites. The introduction of the T allele to Kuyavia 7.4 Ka BP by dairying LBK people is not likely, as suggested by the obtained data. It has not been found in any of Neolithic samples dated between 6.3 and 4.5 Ka BP. The identified frequency profile indicates that both the introduction and the beginning of selection could have taken place approx. 4 millennia after first LBK people arrived in the region, shifting the value of LP frequency from 0 to more than 0.8 during less than 130 generations. We hypothesize that the selection process of the T allele was rather rapid, starting just after its introduction into already milking populations and operated *via* high rates of fertility and mortality on children after weaning through life-threatening conditions, favoring lactose-tolerant individuals. Facing the lack of the T allele in people living on two great European Neolithization routes, the Danubian and Mediterranean ones, and based on its high frequency in northern Iberia, its presence in Scandinavia and estimated occurrence in Central Poland, we propose an alternative Northern Route of its spreading as very likely. None of the successfully identified nuclear alleles turned out to be deltaF508 CFTR.

## Introduction

Approximately 35% of adult people around the world digest lactose after weaning. In most Europeans [1] the so-called lactase persistence/non-persistence (LP/L-nP) is associated with a single nucleotide polymorphism (SNP) C>T located 13910 bp upstream (rs4988235) from the start codon of lactase-phlorizin hydrolase (LPH), within intron 13 of *MCM6* (minichromosome maintenance complex component 6) [1]. The homozygous LCT-13910C/C variant is related to hypolactasia, while the dominant LCT-13910*T allele is responsible for LP [2]. Abundance of the trait and frequency of coding alleles depends on geographic region. In Northern Europe, the enzyme is active in about 90% of adults (even 98% on British Islands [3]), while in southern regions of Europe it falls to approx. 10% [4–7]. Such specific distribution of the trait does not imply the place of its origin and does not facilitate the identification of possible selection conditions and agents. Using two different methodologies, the age of the LCT-13910*T allele was estimated to 2188–20 650 years [8] and 7450–12 300 years [9]. Thus, one can assume that the origin of the allele predates the Neolithization process and cattle domestication in Neolithic Europe [10], which means that, much later, milk could have played a role in its selection and spreading, as many authors suggest [2,11,12]. So far, however, no traces of the allele have been found in Neolithic skeletal material from two main routes, the Danubian and the Mediterranean one, along which first farmers were spreading the new technology [13–15].

Numerous data on LP in modern human populations [5,16] were used to simulate its spatiotemporal distribution profile [2,17], however, it is obvious that only direct information on the LCT-13910*T allele’s occurrence in the past will verify hypotheses and clarify its evolutionary history. Palaeogenetic studies providing information on the allele’s frequency in populations living in various regions and the same period or in the same region during a long time provide an opportunity to estimate its moment of introduction and beginning of selection, possible mechanism operating on the frequency profile and likely agents of selection, as well as the dynamics of changes.

We present, for the first time, LCT-13910*T data collected from a period covering about 250 generations of people living in the same region within a small area of radius of approx. 60 km, belonging mostly to Kuyavia and in part to the neighboring Chełmno land. Data cover a time span of approx. 6 millennia between the Middle Neolithic and the Late Middle Ages, and allow to assess a likely time frame of the T allele’s introduction together with the beginning of its selection. Moreover, we speculate on a mechanism of the T allele’s selection and an alternative route of its spreading.

Below presented are the data on LCT-13910 C>T polymorphism related to LP against variability of HVR-I mtDNA haplotypes identified in the studied individuals to confirm the continuity between populations, relationship between the individuals, their origin and authenticity of the analyzed sequences. The analysis is a part of our research on the reconstruction of Polish prehistoric and historic gene pool, which until now is represented only by a few alleles predisposing to diseases in medieval times [18–20].

## Material and Methods

### Sample information

The samples were indexed as in tables presented in the S1 File. Each number encodes the id of a grave and the name of an archaeological site.

The studied skeletal material is deposited in the co-authors’ places of employment, except for skeletons from Śródka which are taken care of by the Laboratory of Archaeology and Conservation, Henry Klunder, Poznań, Poland. No permits concerning the skeletal material were required for the described study.

### Ancient samples

**Fig 1:**
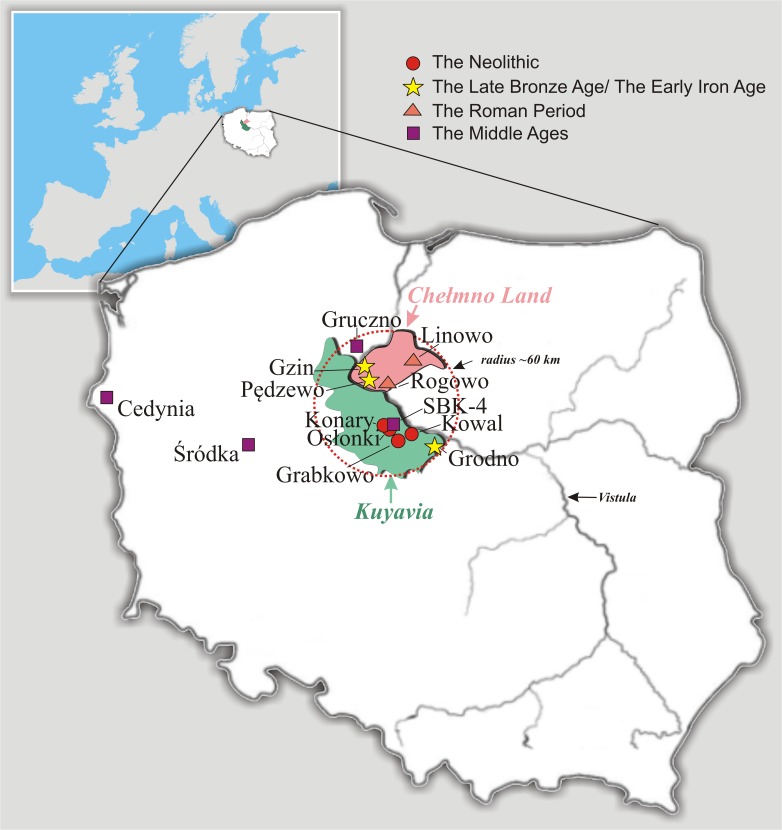
Location of the explored Polish archaeological sites.

Teeth from 231 individuals living in different periods between the Middle Neolithic and the Late Middle Ages were collected. 131 individuals provided HVR-I mtDNA amplifiable sequences, including 80 medieval ones, 34 from the Roman period, 8 from the Late Bronze/Early Iron Age and 9 Neolithic ones. LCT-13910C>T sequence was identified in all except 6 medieval individuals and 3 from the Roman period. The yield of DNA isolation procedure at each archaeological site is presented in Table A in S1 File. The studied samples originated from four medieval sites (Stary Brześć Kujawski-4/14 specimens, Gruczno/15, Cedynia/35, Śródka/16), two representing the Roman period—Wielbark culture (Rogowo/21, Linowo/13), 8 from the Late Bronze Age/Early Iron Age—Hallstatt culture (Gzin/6, Pędzewo/1, Grodno/1) and 9 from Neolithic sites (Grabkowo/4, Kowal/1, Osłonki/1—local Globular Amphora culture; Konary/1, Osłonki/2—Lengyel culture). Data from Cedynia and Śródka are used as a reference for medieval sites. Both these sites are of quite short history (Cedynia 1.2–1.1 Ka BP, Śródka 1.0–0.9 BP) and are located outside Kuyavia/the Chełmno land [21,22]. Cedynia lies at the western border of today’s Poland, approx. 400 km north-west, while Śródka 160 km west from the region. SBK-4, as well as one of the sites dated to the Late Bronze Age/Early Iron Age, i.e. Grodno, together with all the studied Neolithic sites, are closely situated within an area approx. 20 km in diameter in the center of Kuyavia. Other sites, i.e. medieval Gruczno, two remaining sites of the Hallstatt culture, i.e. Gzin and Pędzewo, as well as both sites from the Roman period (Wielbark culture), are all located within a short distance from each other, mostly in the area belonging to the Chełmno land today. All studied archaeological sites are situated within the area of approx. 120 km in diameter (Fig. 1). Burials from the Middle Ages (1.0–0.6 Ka BP) and the Roman period (1.8–1.7 Ka BP) were dated according to the graves’ equipment, while the age of the Neolithic skeletons was estimated with radiocarbon dating (Table B in S1 File). In the case of Hallstatt samples, dendrochronological dating, based on wooden constructions which formed stratification and cultural context, was employed (Table B in S1 File).

To avoid complications implied by kinship, specimens were chosen randomly from distant locations within a given graveyard.

### Extraction of aDNA

In all cases, the withdrawn teeth were placed in a sterile container, delivered to aDNA laboratory at the Department of Molecular Biology, Medical University of Lodz, Poland, and frozen until the beginning of the isolation procedure. After mechanical cleaning (Dremel) each tooth was washed in NaClO for 30 min. in order to remove surface contamination, which was followed by intensive rinsing in 96% ethanol. After exposition of each side to UV light for 30 min., the tooth was ground in a freezer mill (SPEX SamplePrep 6770) and 0.5 to 0.9 g of the toothpowder was decalcyfied in 0.5 M EDTA (pH = 8.0) for 48 hrs. Proteinase K and *N*-phenacyltiazolium bromide (PTB) were added to DNA solution and incubated at 56°C for further 2 hrs to degrade DNA-associated proteins and remove cross-links. Subsequently, the obtained solution was submitted to DNA isolation in MagNA Pure Compact Nucleic Acid Purification System (Roche) as guided by the manufacturer. Obtained DNA was quantified prior to its amplification (Qubit 2.0, Invitrogen or Eco Real-Time PCR System, Ilumina). Isolation of DNA in a closed automatic system and using not more than 8 samples at a time prevented against batch-effects. Appropriate mock controls with ready-to-use chemicals were performed. Samples processed at the same time originated from the same archaeological site. Isolation of samples from different sites was processed in various periods depending on time of their acquisition. In almost all cases samples from one archaeological site were obtained at the same time with exception of Neolithic and Hallstatt ones which were processed separately.

### LCT-13910C>T genotyping

A DNA fragment spanning the sequence of the LCT-13910C>T variant was amplified with the primer pair 5’-GCGCTGGCAATACAGATAAGATA-3’ and 5’-AATGCAG GGCTCAAAGAACAA-3’, yielding 111 bp PCR product. Amplification was performed in 25 μl, including 3–4 μl of sample extract, in the presence of all standard reagents, including AmpliTaq Gold (Applied Biosystems), at the annealing temperature of 55°C, during 38 cycles. After purification on spin columns (Clean-up, A&A Biotechnology) amplicons were extended using BigDye 3.1 termination-ready reaction mix (Applied Biosystems). Each sequencing reaction mixture (20 μl) contained 4 μl of BigDye mix, 30 ng of primer and 50–70 ng of amplicon. Initial denaturation at 95°C for 5 minutes was followed by 36 cycles at 95°C for 30 seconds, 56°C for 8 seconds, and 60°C for 4 minutes. Extended products were purified on spin columns (ExTerminator, A&A Biotechnology), dried in a Speed-Vac system (Savant), resuspended in 20 μl of deionized formamide and sequenced on ABI Prism 3130 Genetic Analyzer (Applied Biosystems). Sequences were edited and analyzed using BioEdit and MEGA 4 software [23].

### Genotyping of the most frequent pathologic allele of the CFTR gene

Wild and mutated alleles (CFTR/delta F508 CFTR) were amplified using KAPA HRM Fast PCR Kit (Kapa Biosystems). A 3-bp difference between physiological and pathological alleles was estimated by HRM (High Resolution Melting) method on Eco Real-Time PCR (Illumina).

### Mitochondrial DNA analysis

Two primer pairs, L16112 (5’-CGTACATTACTGCCAGCC-3’) and H16262 (5’-TGGTATCCTAGTGGGTGAG-3’) as well as L16251 (5’-CACACATCAACTGCAACTCC-3’) and H16380 (5’-TCAAGGGACCCCTATCTGAG-3’) were applied to amplify the HVR-I between 16112 and 16380 bp. Obtained product was usually readable between 16115 and 16340 bp as two overlapping PCR products of 186 and 171 bp. HVR-I amplification and sequencing parameters were comparable to those applied during PCR of the *MCM6* gene, except the annealing temperature: 54°C.

### Indirect estimation of aDNA preservation [24]

Pre-selection of each tooth was followed by its grinding and incubation of the obtained powder in 1 M HCl (300 mg in 5 ml of HCl) at 48°C for 5 hours. The soluble fraction was then separated from insoluble collagen (7000 x g, 5 min). After a few washings (until neutral pH was reached) samples were dried at 56°C for 18 hours. The amount of collagen was then calculated as the ratio of dry weight of insoluble fraction to initial weight of tooth powder.

### Contamination control and authentication of DNA sequences

Analysis of DNA from human remains faces a number of methodological problems such as contamination, post-mortem chemical damage and limited availability of endogenic DNA. The preparation step and molecular analysis were carried out in a laboratory specially dedicated to work with ancient DNA, which never witnessed molecular analysis of modern molecules. Cleaning and powdering of skeletal material, as well as DNA extraction and its amplification, were carried out by personnel wearing protective disposable clothes. All operations were conducted under laminar flow hood (Heraeus Biohazard II) using DNA-free disposables equipped with a filter (Sarstedt). Decontamination with DNA-ExitusPlus (AppliChem) solution of every instrument and lab surface after each experiment and UV irradiation of clean room until the next activity was a routine. Multiple mock controls were implemented at each step of the procedure. Verification of authenticity of the analyzed DNA fragments was performed through identification of mtDNA sequence patterns of lab personnel involved in processing of the samples and comparison with sample DNA. Our Personal Genetic Identification Database (PGID) consists of mtDNA haplotypes and allelic variants of a few genes, including *MCM6*. Such multiparameter profile of individual patterns provides information for precise recognition of contaminating staff member, if any. Lab staff in the Department of Molecular Biology, Medical University of Łódź, working with human ancient DNA exhibit rather rare haplotypes, easily recognizable due to individual mutations (e.g. hg C with 16297C/16223T/16327T, hg U5 with 16189C/16270T/16291T, hg K with 16284G/16319A, and others). DNA was extracted from two teeth of a specimen, each powdered independently, from at least two different powder portions. Teeth from one individual were processed by lab workers of different PGID profile. We rejected laborious and expensive cloning and decided to sequence multiple isolates from the same specimen, as suggested by Winters et al. [25], successfully applied by us [26] and others [27]. In most cases, 4 isolates provided consensus sequence (2 from each of two separate teeth) of every individual’s DNA. Additional tooth analysis was not necessary, except cases of low initial copy number due to skeletal material’s degradation. Loss of repeatability resulted in rejection of a sample from further analysis and the procedure was repeated using another tooth, if available.

### Statistical analysis

The analysis of mtDNA HVR-I sequence was performed using Arlequin 3.5 [28], while HaploGrep database was used for identification of haploroups [29]. Genetic differentiation of the studied populations or the distance between them (*F*_*ST*_) was estimated according to the formula of Reynolds, *P*-values resulting from 10,000 permutations. Statistics of the frequency of LP genotype was calculated using Microsoft Excel with GenAlEx 6.4 platform [30] and the T allele differences were assessed by the Fisher exact test. Multiple testing was accommodated with Bonferroni correction. Confidence intervals for the T allele frequency were calculated according to the method of Fung and Keenan allowing for small sample and population size, as well as deviation from HWE [31]. Confidence intervals for the trait frequency, on the other hand, with the exact method using the hypergeometric distribution. The probability level *P* < 0.05 was considered in all calculations as statistically significant. For the purpose of modelling no spatial structure was introduced to the calculations and the data from different time points were treated as representing a single population (see Material and Methods for details on the distribution of archaeological sites). Key dates considered during calculations of time of the T allele introduction and beginning of lactase persistence selection in the region of Kuyavia and the Chełmno land are presented in Table L in S1 File.

When dealing with human ancient DNA, the size of considered populations might be significant, because of possible contribution of genetic drift. In the following analysis the effective population size is taken to change deterministically according to the generalized logistic function:

$$N\left[t\right]=A+\frac{K-A}{{\left[1+Qe^{-B\left(t-M\right)}\right]}^{\frac{1}{\nu }}}$$

were *A* = 80, *K* = 450, *B* = 0.0146, *Q* = 1, *M* = − 233, *ν* = 0.001 (see S1 Fig. for plot). The curve gives the size of 80 for the Neolithic populations, 150 for Roman and 300 for medieval ones [32], which indeed produces considerable drift. In all cases, the effects of demographic and environmental fluctuations are neglected.

Consequently, the stochastic Wright-Fisher model was employed. A numerical scanning was performed to estimate a probable time of allele’s introduction and test for possible selection. Frequency curves of the T allele were generated with the help of binomial distribution with number of trials equal to doubled population size and probability of success (finding of the T allele) calculated from the formula:

$$p\left[t+1\right]=\frac{w_{\text{TT}}\;p^{2}\left[t\right]+w_{\text{TC}}\;p\left[t\right]q\left[t\right]}{w_{\text{TT}}\;p^{2}\left[t\right]+2\;w_{\text{TC}}\;p\left[t\right]q\left[t\right]+w_{\text{CC}}\;q^{2}\left[t\right]}$$

Here, *p*[*t*] denotes the T allele frequency in generation *t* just after random mating (similarly, *q*[*t*] for the C allele), *w*_*ij*_ the fitness of particular genotype, assumed to be constant through evaluation time, and *p*[*t*] + *q*[*t*] = 1, because the considered gene can be considered bi-allelic as we did not find in the studied material any other SNP responsible for lactase persistence (see Results). Since natural selection shouldn’t be sensitive to the difference between genotypes containing dominant T allele and we are interested in relative fitness, it was assumed that *w*_TT_ =*w*_TC_ = 1. Moreover, we define the selection coefficient as *s =* 1 − *w*_CC_. Based on the above, curves for lactose-tolerant phenotype were generated by applying the formula

$$LP\left[t\right]=\frac{p{\left[t\right]}^{2}+2p\left[t\right]q\left[t\right]}{p{\left[t\right]}^{2}+2p\left[t\right]q\left[t\right]+\left(1-s\right)q{\left[t\right]}^{2}}$$

where *LP*[*t*] denotes the trait frequency in generation *t* (we used this formula since it was in acceptable agreement with random pairing of alleles forming the genotypes, see S2 Fig., but easier to implement numerically). That is, we assume that the only force significantly disturbing the HWE was natural selection.

The range of selection coefficient *s* was 0 to 0.1, while the T allele introduction time *t*_0_ 7800 BP to 2700 BP, the former scanned in steps of 0.005, the latter taken every 10 generations (generation = 25 years). A low initial value of the T allele frequency equal to 0.05 was assumed in each case.

**Fig 2:**
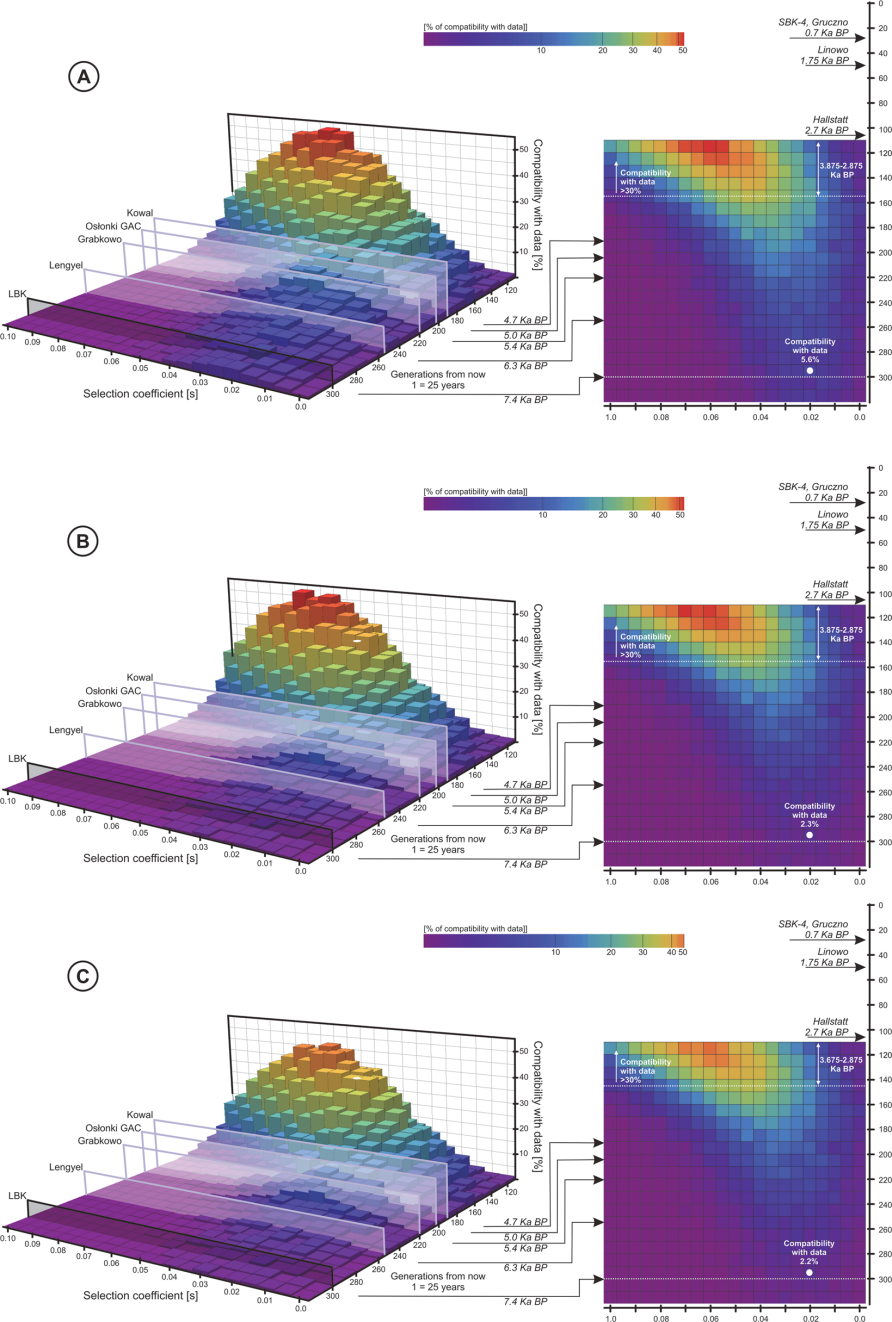
Each 3D plot together with its 2D projection presents probability of a scenario with given selection coefficient and the T allele introduction time, assuming variable population size (see S1 Fig. for the shape of the size curve). The height/color of each bar represents the percentage of Wright-Fisher curves (number per 1000) falling into appropriate confidence intervals shown in Table 2: A—the T allele, B—LP, C—the T allele and LP jointly.

**Fig 3:**
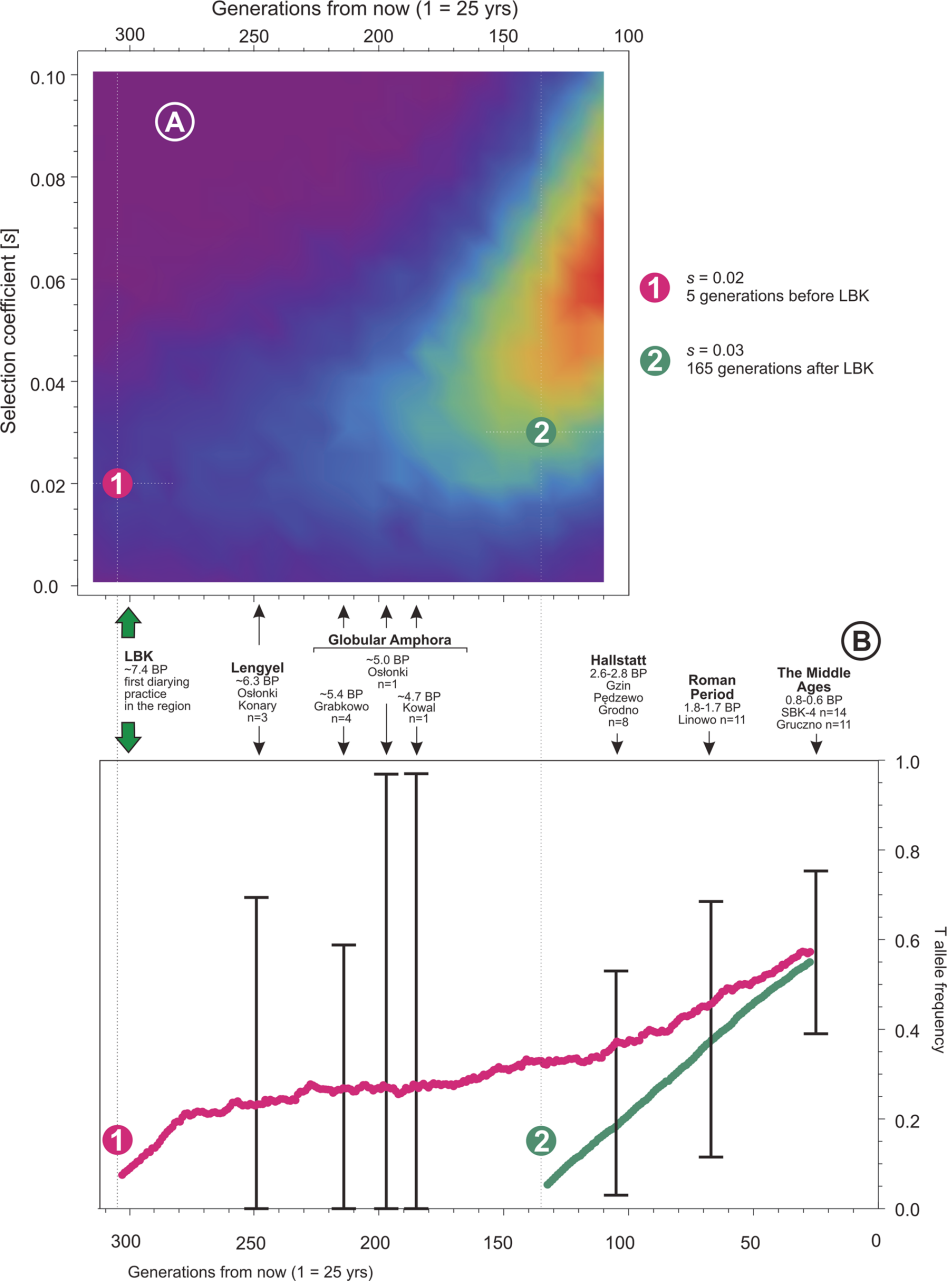
Mean allele frequency calculated for two chosen scenarios from Fig. 2A (only curves falling into confidence intervals were taken into account) along with their probabilities marked on the interpolated version of 2D part of Fig. 2A (curve 1—probability 0.027, curve 2—probability 0.297).

The criterion for choosing a value of selection coefficient and the T allele introduction time as significant was that they noticeably increased the percentage of the allele and the trait frequency curves falling into appropriate confidence intervals. This procedure distinguishes a whole set of these parameters, not only a single pair (Fig. 2 and Fig. 3). Moreover, observed were small fluctuations in percentage between different runs of numerical calculations (of the order of a few percent).

## Results

The amount of collagen in chosen specimens showed various degree of biomolecules’ preservation at different graveyards and archaeological sites, being as high as 5.9 ± 1.8% in Cedynia where remains were deposited in marl soil, and as low as in SBK-4–3.5 ± 1.7%, clearly differentiating DNA yield.

### LCT-13910*T

The prevalence of LCT-13910*T and LP genotype were significantly different in specimens found at each of the studied archaeological sites (Table 1, Table 2, Table 3, Fig. 1). The T allele frequency differed even between studied medieval sites, being much higher in SBK-4 (0.5) and Gruczno (0.64) than in contemporaneous Cedynia (0.2) and Śródka (0.29). Considerably different from that of modern inhabitants of Poland (0.3) [33] was the distribution of the T allele in SBK-4 (*P* = 0.025, *F*_*ST*_ = 0.06) and Gruczno (*P* = 0.0002, *F*_*ST*_ = 0. 118), in contrast to values estimated for Cedynia (P = 0.163, *F*_*ST*_ = 0.012) and Śródka (*P* = 0.487, *F*_*ST*_ = 0.0008). Having in mind high frequency of lactase persistence in people from medieval SBK-4 (0.86) and Gruczno sites (0.82), we determined the frequency of the T allele and LP in individuals from two small populations living in Rogowo and Linowo a millennium earlier, both representing the Roman period (Wielbark culture; 1.8–1.7 Ka BP) in the studied region. LCT-13910*T was also found in these samples, however, at a lower frequency than in SBK-4 and Gruczno (Rogowo—0.525/LP = 0.7 and Linowo—0.35/LP = 0.6).

**Table 1:** Distribution of LCT-13910C>T in individuals from Polish archaeological sites and modern Polish and Scandinavian populations.

LCT -13910 genotype		Hallstatt n = 8	Roman period Linowon = 11	Roman period Rogowo n = 20	Middle Ages Gruczno n = 11	Middle Ages SBK-4 n = 14	Middle Ages Cedynia n = 35	Middle Ages Śródka n = 14	Modern Poland n = 223[16]	Modern Scandinavian = 1622 [1, 81]
**C/C**		6	4	6	2	2	21	8	109	162
**C/T**		1	6	7	4	9	14	4	96	535
**T/T**		1	1	7	5	3	-	2	18	925
**T allele frequency**		0.19	0.36	0.525	0.64	0.54	0.2	0.29	0.3	0.73
**LP frequency**		0.25	0.64	0.7	0.82	0.86	0.4	0.43	0.51	0.90
**HWE (P>)**		0.09	0.55	0.18	0.48	0.27	0.14	0.26	0.97	0.88
**Against modern Poles**	*F*_*ST*_	0.015	0.006	**0.055**	**0.118**	**0.06**	0.012	0.00008	-	
	*P*	0.228	0.715	**0.00068**	**0.0002**	**0.025**	0.163	0.487	-	
**Against modernScandinavians**	*F*_*ST*_	**0.194**	0.066	0.009	0.0003	0.007	**0.182**	**0.113**	**0.107**	-
	*P*	**1.22x10** ^**−8**^	0.079	0.332	0.599	0.327	**9.17x10** ^**−8**^	**3.64x10** ^**−8**^	**2x10** ^**−13**^	-

**Table 2:** Confidence intervals for the T allele and LP frequency found at studied archaeological sites and included in numerical calculations.

**LCT -13910 genotype**	**Neolithic Lengyel**	**Neolithic Grabkowo**	**Neolithic GAC Osłonki**	**Neolithic Kowal**	**Hallstatt**	**Roman period Linowo**	**Middle Ages Gruczno**	**Middle Ages SBK-4**
	**n = 3**	**n = 4**	**n = 1**	**n = 1**	**n = 8**	**n = 11**	**n = 11**	**n = 14**
**T allele frequency**	0	0	0	0	0.19	0.36	0.58	
**Fung-Keenan 95% C.I.**	[0, 0.694]	[0, 0.588]	[0, 0.969]	[0, 0.97]	[0.03, 0.53]	[0.115, 0.685]	[0.39, 0.753]	
**LP frequency**	0	0	0	0	0.25	0.64	0.84	
**Exact hypergeometric 95% C.I.**	[0, 0.688]	[0, 0.588]	[0, 0.963]	[0, 0.963]	[0.04, 0.64]	[0.315, 0.883]	[0.65, 0.95]	

**Table 3:** The value of the Fisher exact test.

	**Modern Poland**	**Śródka**	**Cedynia**	**SBK4**	**Gruczno**	**Linowo**	**Rogowo**
**Modern Poland**	-						
**Śródka**	0.9999	-					
**Cedynia**	0.1412	0.1881	-				
**SBK4**	**0.0009**	**0.0005**	**0.0000**	-			
**Gruczno**	**0.0000**	**0.0000**	**0.0000**	0.1955	-		
**Linowo**	0.4522	0.3650	0.0177	0.0154	**0.0001**	-	
**Rogowo**	0.0024	**0.0014**	**0.0000**	0.8873	0.1147	0.0323	-
**Hallstatt**	0.0995	0.1357	0.9999	**0.0000**	**0.0000**	0.0109	**0.0000**

Comparison of the T allele frequency found at studied archaeological sites. Statistically significant differences after Bonferroni are typed in boldface.

Frequent cremation practices in the Bronze Age resulted probably in paucity of remains at archaeological sites of the period, thus we studied only 8 individuals dated to the turn of the Late Bronze and the Early Iron Age, representing the Hallstatt culture. They were found at three different neighboring sites (Gzin/6, Pędzewo/1 and Grodno/1), and the obtained results were pooled to calculate the allele frequency—0.19 (LP = 0.25). In contrast, none of 9 Neolithic specimens carried the LCT-13910*T allele, as has been observed also for Central and Southern European Neolithic samples [13–15]. Six of them were unearthed very close to SBK-4, i.e. in Osłonki /1, or not farther than 20 km south-east, in Kowal/1 and Grabkowo/4, all ^14^C-dated between 5.5 and 4.5 Ka BP. They belonged to a local Globular Amphora culture. Three others were ^14^C-dated to 6.5–6.1 Ka BP and represented Brześć Kujawski Lengyel culture (BKLC) in Osłonki/2 and Konary/1. For details see Table B in S1 File.

Sequencing of intron 13 of the *MCM6* gene in 131 ancient individuals indicated that LCT-13910C>T is probably the only SNP responsible for the regulation of lactase activity in Polish population, since none of other known SNPs (LCT-13907C/G, LCT-13909T/A, LCT-13913T/C, LCT-13915T/G) was found among the studied samples.

### Introduction and the beginning of the LCT-13910*T allele selection

The employed model showed that the closer from the arrival time of LBK to the Hallstatt period, the more probable is the introduction of the T allele and participation of the selection process in its sustaining in population. That is, moving towards the present times from 7.4 Ka BP and allowing for non-zero selection coefficient one is able to increase the mentioned probability of the T allele introduction and selection from several to about 50%. However, approaching too close to Hallstatt resulted in rather high selection coefficients, reaching the value of 0.06. To allow for a wide range of values and significant probability of scenarios (probability >30%) one obtains a lower bound for the T allele introduction time equal to approx.145 generations after the arrival of LBK people (Fig. 2A). Although the probability results for sole LP (Fig. 2B) and LP verified against data together with T (Fig. 2C) differ by a few percent, they do not change the final conclusion. Fig. 3 illustrates the applied calculation method.

### mtDNA

Identified HVR-I mtDNA haplotypes and haplogroups clearly suggest differences in profile of the studied groups, as presented in Table 4 and Tables D-K in S1 File. We used rCRS description to cover main European haplogroups (H+U+U4+J) which are indistinguishable if haplogroup identification is based only on HVR-I sequence. None of mtDNA haplogroups characteristic for foragers was found in individuals from the Neolithic and Rogowo, in contrast to the remaining younger populations. *F*_*ST*_ values confirmed discontinuity between the Rogowo and other populations (Table 5), suggesting its fundamentally different origin in the maternal lineage, which made the authors reject the population from further considerations, despite high abundance of LP (0.7), related probably to the population’s origin. Mesolithic haplogroup U5b1b1(0.125) was identified only among people representing the Hallstatt culture (Table E in S1 File) and dated to 2.8–2.6 Ka BP. U5a1d2a amounted to 0.077 among people living in Linowo (Table F in S1 File). In medieval samples, U5b1d and U5a amounted to 0.214 in SBK-4 (Table I in S1 File), U5a2a (0.133) was found in Gruczno (Table H in S1 File), U5b1d, U5a and U5 (0.143) in Cedynia (Table J in S1 File) and U5a, U5 (0.25) in Śródka (Table K in S1 File).

**Table 4:** Identified haplogroups and their contribution to each of the studied populations.

**Haplogroup**	**Neolithic**	**Hallstatt**	**Linowo**	**Rogowo**	**Gruczno**	**SBK4**	**Cedynia**	**Śródka**	**Total ancient**	**Modern Poland**
**(%)**	**n = 10**	**n = 8**	**n = 13**	**n = 21**	**n = 15**	**n = 14**	**n = 35**	**n = 16**	**n = 132**	**n = 436**
**H+U+U4+J**	**7**	**5**	**7**	**19**	**9**	**9**	**21**	**11**	**88**	**253**
	(70)	(62.5)	(53.8)	(90.6)	(60)	(64.3)	(60)	(68.7)	(66.7)	(58)
**U5**	-	**1**	**1**	-	**1**	**3**	**5**	**4**	**15**	**38**
		(12.5)	(7.7)		(6.6)	(21.4)	(14.3)	(25)	(11.4)	(8.7)
**K**	**2**	**1**		**1**	**1**	**1**	**3**	-	**9**	**15**
	(20)	(12.5)	-	(4.7)	(6.6)	(7.1)	(8.5)		(6.8)	(3.4)
**T**	**1**	**1**	**4**	-	**2**	-	**3**	-	**11**	**41**
	(10)	(12.5)	(30.8)		(13.6)		(8.5)		(8.3)	(9.4)
**U2**	-	-	0	0	**1**	-	**1**	-	**2**	**4**
					(6,6)		(2.9)		(1.5)	(0.9)
**HV0**	-	-	0	**1**	**1**	**1**	**1**	**1**	**5**	**21**
				(4.7)	(6.6)	(7.1)	(2.9)	(6.3)	(3.7)	(4.8)
**I**	-	-	**1**	-	-	-	-	-	**1**	**8**
			(7.7)						(0.8)	(1.8)
**Z**	-	-	-	-	-	-	**1**	-	**1**	-
							(2.9)		(0.8)	

**Table 5:** Continuity between the studied populations based on HVR-I haplotypes and calculated as fixation index (*F*_*ST*_).

	Modern Poland	Śródka	Cedynia	SBK4	Gruczno	Linowo	Rogowo	Hallstatt
**Modern Poland**	-							
**Śródka**	0.023*	-						
**Cedynia**	0.010*	0.015	-					
**SBK4**	0.032	0.028	0.017	-				
**Gruczno**	0.008	0.000	0.000	0.031	-			
**Linowo**	0.005	0.022	0.029	0.073*	0.003	-		
**Rogowo**	**0.036** *	**0.145** ***	**0.089** ***	**0.142** *	**0.129** **	**0.099** *	-	
**Hallstatt**	0.000	0.005	0.000	0.019	0.000	0.000	**0.074** *	-
**Neolithic**	0.000	0.054	0.000	0.056	0.000	0.000	**0.109** *	0.000

*P < 0.05;

**P < 0.001;

***P < 0.0001

## Discussion

One should keep in mind that a sequence to be isolated from fossil material and analyzed is frequently difficult to access, both due to degradation of molecules and, in our case, limited area covered by the study. Success in the analysis of fossil material depends mostly on the degree of chemical alteration of DNA structure, which in turn depends on features of the surrounding environment. Sometimes location of samples and their dating suit the purpose of a project, however, high degradation degree of isolated DNA fragments, if they survive at all, results in lack of PCR products or samples are simply not available due to cultural processes, as was in the case of cremation practices. One should also remember that ancient populations were much less numerous than modern ones, which severely limits the chance to obtain hundreds of samples for an analysis. On the other hand, this last feature has a positive aspect—a smaller experimental sample is more representative for the whole population. Some methods exist that allow to estimate this feature, e.g. by knowing the number of burials, average life expectancy and predicted duration of cemetery use. For instance, it has been found in the case of the Rogowo population (unfortunately, rejected from calculations), that at least 288 individuals have been buried in inhumation and cremation [34] and the graveyard was used for approx. 150–200 years. It means that isolation of amplifiable sequences from 20 individuals we have studied reflects more than 6% of the whole population living at Rogowo and almost half of the average number of individuals living at the same time (∼50 individuals). Nevertheless, the data for application of such methods, if available, are often uncertain. The above means also that there is much higher probability to find the same haplotype in a smaller graveyard than in a bigger one as we observed in the case of Rogowo (Table G in S1 File). Since the population size might significantly influence the genetic drift, we treat many aspects of statistical analysis in this work more as a source of suggestions than as a tool for obtaining confirmation of particular hypotheses.

### Authenticity of the analyzed sequences

Risk of contamination with exogenous DNA is one of the major limitations in human ancient DNA studies, even strengthened when the classical PCR approach applied. In order to maintain the highest possible degree of authenticity of isolated sequences, we have combined some of the suggested criteria [35] and our own approach: replication of obtained data, multisequencing applied instead of cloning, screening for mtDNA of people involved in acquisition and analysis of samples were the main elements of the procedure. High diversity found in isolated and analyzed mitochondrial and genomic sequences imply their authenticity, indicating appropriate treatment and the highest possible effective protection against contamination with exogenous DNA molecules. Otherwise, limited types of changes would dominate the distribution of identified haplotypes (the number of identical haplotypes found among analyzed specimens is highly limited, Tables D-K in S1 File). Two out of 14 haplotypes from SBK-4, which belong to haplogroup H, carried the same mutation 16234T, but only one of them was lactose tolerant. Although three individuals out of 35 from the Cedynia graveyard carried the same changes 16224C and 16311C in HVR-I (hg K), two of them were of different LP genotype, and it is likely that HVR-II sequencing would show other mutations, since they were buried distantly from each other. Similarly, at the Rogowo site three out of 20 studied individuals carried the same change 16189C indicating hg H1. Two of them carried the same LP haplotype which resulted in rejection of one from further considerations as a potential family member. Nevertheless, we did not observe any significant change of frequency (from 0.7 to 0.69).

Moreover, none of the haplotypes identified in 131 specimens corresponded to any of the haplotypes assigned to staff involved in the excavation process, DNA isolation and molecular analysis of the samples (Table C in S1 File). The reliability of the isolation result is improved also by the identified distribution of the LCT-13910C/T alleles, which varied between studied ancient populations and allowed to distinguish them easily from each other as well as the modern one.

We also assumed that cytosine deamination has not influenced the obtained results, since the C allele involved in miscoding lesions occurs rather on the overhanging ends, while the identified SNP LCT-13910C/T is localized 81 bp from the 3’ and 29 bp from the 5’end. Thus, even if every amplified fragment of ancient DNA was only as long as the PCR product, the probability of deamination would not exceed 1–2% as documented by Biggs *et al*. [36], since only a few nucleotides from 3’ end are prone to C→U deamination [37]. Moreover, the possibility of finding the result of C→T transformation decreases significantly with each subsequent sequencing in case when consensus sequence is obtained, as suggested by Winters *et*. *al*. [25]. In our case an even more rigorous strategy was applied. Instead of using the same DNA isolate [25], we performed multiple sequencing of DNA isolated from different teeth of each studied individual, a methodology successfully applied by us earlier [26].

### HVR-I mtDNA

**Fig 4:**
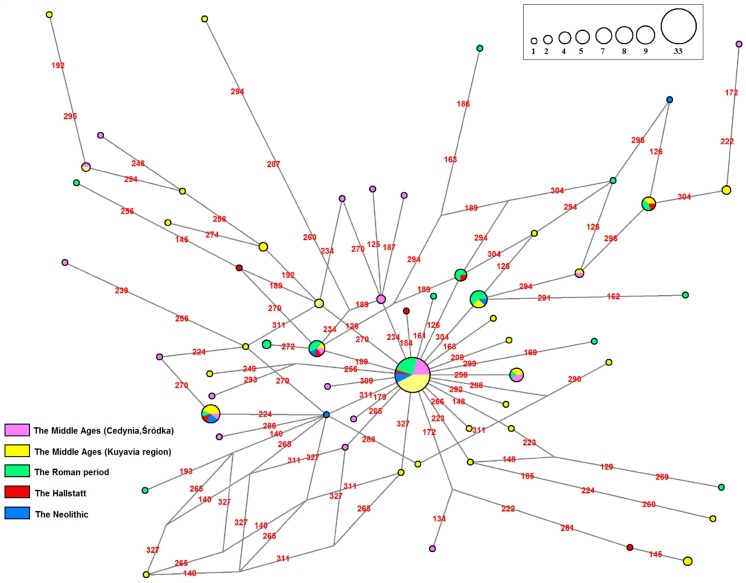
Median joining phylogenetic network of 131 ancient inhabitants of Polish lands based on HVR-I sequences (nt 16115–16340, motifs in red). 80 samples are from archaeological sites located on a relatively small area belonging to Kuyavia and the Chełmno land and represent people living between 6.5–6.1 Ka BP and 0.8–0.6 Ka BP, i.e. 9 individuals dated to Polish Neolithic (3—Lengyel culture, 6—Globular Amphora culture), 8 from Polish Late Bronze Age/Early Iron Age (Hallstatt culture), 34 from Polish Roman Period (Wielbark culture; Linowo—13, Rogowo—21) and 29 from Polish Middle Ages (Gruczno—15, SBK-4–14). Additional 51 medieval samples collected outside Kuyavia and the Chełmno land (Cedynia—35, Sródka—16) constituted the reference group. Origin of the sample is marked with different colors. The size of the node is proportional to the number of individuals.

Having an opportunity to sample and characterize a large number of individuals living over several millennia in the same region, not encountered in the literature so far, we followed the HVR-I sequence to evaluate genetic continuity, heterogeneity, putative origin and their relationship to ancestral and descendant populations. Based on HVR-I sequence and comparative haplotype analysis, it can be demonstrated that, except the subpopulation from Rogowo, all studied samples share continuity in the maternal lineage with an ancestral population (Table 5). A sign of the interaction between first farmers and foragers, i.e. the presence of hg U5b, within the studied samples was found only in the Hallstatt group (2.8–2.6 Ka BP), which does not mean that earlier contacts did not take place (Table E in S1 File). U5/U5a/U5b, most abundant haplogroups in the Mesolithic Europe [38–40], were also identified, however, in populations living later, as presented in Tables F-K in S1 File. The presence of haplogroup K, which arose 31.4 Ka ago somewhere between Near East and Europe [41] and was highly abundant across the Neolithic Europe [13,39], confirms a contribution of first farmers’ substrate to the maternal lineage of the region from the Neolithic through medieval times (Tables D-K in S1 File). However, haplotype changes characteristic for hg K and common in LBK individuals [38,40] were not found among three individuals representing the Lengyel culture (Table D in S1 File), unlike, however, in the case of two of six other Neolithic individuals of the Globular Amphora culture. This might suggest a diverse origin of these cultural groups or impact of migrants during the latter period. Overall comparison of the literature data obtained for Mesolithic [38,39], Neolithic [13,39,42] and modern specimens [43] with those obtained herein depicts a gradual decrease of K and increase of U5 frequency during the formation of medieval population. Overall relations between haplotypes identified in 131 ancient inhabitants of Polish lands are presented in Fig. 4 as median joining network.

### Lactase persistence

The number of reports regarding the genotype of LP in prehistoric and historic populations is rather limited, however, the database is still being enriched. Together with demographic expansion of first Neolithic cultures, the frequency of many alleles/traits in the European gene pool underwent significant change differentiating sub-regions as a consequence of natural selection, genetic drift, but also as a result of migrations. LP exemplifies such a trait and is believed to be one of the human phenotypic features which underwent fast frequency increase during only a few thousand years [44–54]. Dairy diet which provides basic biological components, including a source of energy, vitamins, ions and water, was considered by many authors among agents increasing the probability of survival under extreme conditions in the past [2,5]. Archeological findings from south-eastern European sites confirm that milk processing started about 8–9 Ka BP [55] and was practiced across Neolithic cultures.

Having access to skeletal material from people living in successive generations between 6.5–6.1 BP and 0.8–0.6 Ka BP within a small area covering a region with a long history dating back to the Neolithic (modern Kuyavia and the Chełmno land), we attempted to identify the LCT-13910*T allele and estimate its frequency. Unfortunately, the skeletal material from the LBK period providing amplifiable DNA was unattainable. The fact that we did not find the T allele in any of the studied Neolithic samples representing successive populations and dated between 6.5 and 4.5 Ka BP suggests that it might not have been introduced to the region by dairying LBK people entering Kuyavia in the middle of the 8^th^ millennium BP [56,57]. One can expect that the T allele, if present in the LBK population, should be spread, as an advantageous one, between successive generations and selected rapidly in small Kuyavian dairying populations [57], assuming genetic drift weak enough to allow for visible selection. This may have been the case for the population from Neolithic Osłonki, the size of which was estimated at 70–80 individuals [58], a minimum for a human group to be self-sufficient in economic and social terms [59]. So, it can be assessed that milking habits of small population in rather tough conditions, as suggested for the Neolithic [60,61], would favor selection of the T allele immediately after its introduction. In contrast to the nine Neolithic lactose intolerant individuals, already two of eight studied representatives of a much later period, associated with the Hallstatt culture, tolerated lactose, one being homozygous.

The numerical scanning indicates an interval of increased probability of the selection starting point in a range between 3.875 and 2.875 Ka BP (Fig. 2), assuming no significant events affected demography or disturbed the selection process. The result falls well into the period following the one during which profile of mtDNA lineages characteristic for the Early Neolithic LBK was deeply altered, as reported by Brotherton et al. [62]. The obtained result seems to confirm the suggestion, based on the T allele absence in Neolithic samples, that LBK people entering the studied region at ∼7.4 Ka BP [56] have not introduced LCT-13910*T although they were practicing dairying [57]. Obviously, only analysis of further individuals representing different archaeological sites in Kuyavia and the Chełmno land, especially from the Late Neolithic and the Bronze Age, through successive generations, can reveal the true shape of the obtained profile, especially in early stages of selection.

Obtained results of the T allele screening since the Neolithic seem to indicate even more rapid increase in frequency of the T allele than proposed by calculations based on modern data [45,46,63]. LP frequency in the region of Kuyavia and the Chełmno land could have raised from 0 to 0.86 within approx. 110 generations, at least 150–160 generations after the LBK arrival (generation = 25 yrs).

Attempts to establish when and where the selection of the T allele has started are so far based on backward simulation indicating the Hungarian Plain/Carpatian Basin some 7.5 Ka BP [17]. It is believed that LCT-13910*T was carried by first farmers [64], most probably by LBK people [17], although none of the representatives of this culture studied until now could drink fresh milk [14,15]. Limited direct data from other sites and periods since the Neolithic lead to ambiguous conclusions regarding the selection mechanism and routes of the allele’s spreading. The only Neolithic lactase-persistent people found until now lived well after the LBK people entered Central Europe [65], i.e. between 5 and 4.5 Ka BP, in populations representing diverse archaeological cultures. They were identified among the Late Neolithic/Chalcolithic individuals living on the territory of the Basque Country (T = 0.23; LP = 0.27) [66] and in Scandinavia among late hunter-gatherers representing Pitted Ware culture (PWC) (T and LP = 0.05) [67]. Why was the T allele not found in farmers who entered Europe 7.5 Ka BP and had access to milk, the main factor necessary [12] in the selection process, as commonly postulated [12,17]? It is possible that the allele LCT-13910*T was not found thus far neither along the Danubian nor the Mediterranean route since:

- it was present at a very low frequency [14], which could indicate that LBK people did not use fresh milk or
- it was not present in first farmers at all (LBK and Cardinal cultures), possibly being not of the early Neolithic origin, as it was found in Iberia only approx. 5 Ka BP, [66], and 4.5 Ka BP in people of PWC, foragers living in the Gotland Island [67].

### Speculation on the scenario of the T allele’s spreading in Europe

The T allele was not found among LBK people as is evidenced by the data obtained until now [13,14]. This is in accordance with our results, since we did not find it in populations following LBK, i.e. belonging to Lengyel and Globular Amphora cultures (Table D in S1 File), although first farmers had a chance to pass it during at least 6 centuries [56]. Both populations inhabited the same area and used milk introduced to it as early as in the middle of 8^th^ millennium BP, as recently confirmed by the oldest evidence of cheese making in Kuyavia by LBK people [57]. The observed lack of the T allele in the very first as well as in later Neolithic populations indicates its rather distinct than LBK people source and a much later introduction to the Neolithic region of Central Poland.

Two main routes of the Neolithization process are commonly accepted as involved in spreading of new technology: one along the Danube river, the so-called Danubian route leading to Central Europe, and the other along northern coast of the Mediterranean Sea, the so-called Mediterranean route [65]. LCT-13910*T was not found in first farmers six among who were buried on the Hungarian Plain and in Central Germany [14]. Recently, lactose intolerance was found in skeletons of people living approx. 5 Ka BP. They represent the Cardial culture from Trielle, South France, a site located on the Mediterranean route [13]. At almost the same time, approx. 600 km north in the area of modern Basque Country, there lived a population of which as many as 27% were lactose tolerant [66]. If not delivered by the two main routes, the allele could have been already present in the Mesolithic population ancestral to modern Basques or could have been introduced to Iberia, e.g. by people coming from Africa through the Strait of Gibraltar. In modern-day Moroccans and living nearby Saharawi, the T allele is present at a rather moderate frequency, i.e. 0.21 and 0.26, respectively [4]. However, it should be mentioned that in case of both populations the T allele is not the only one responsible for LP.

**Fig 5:**
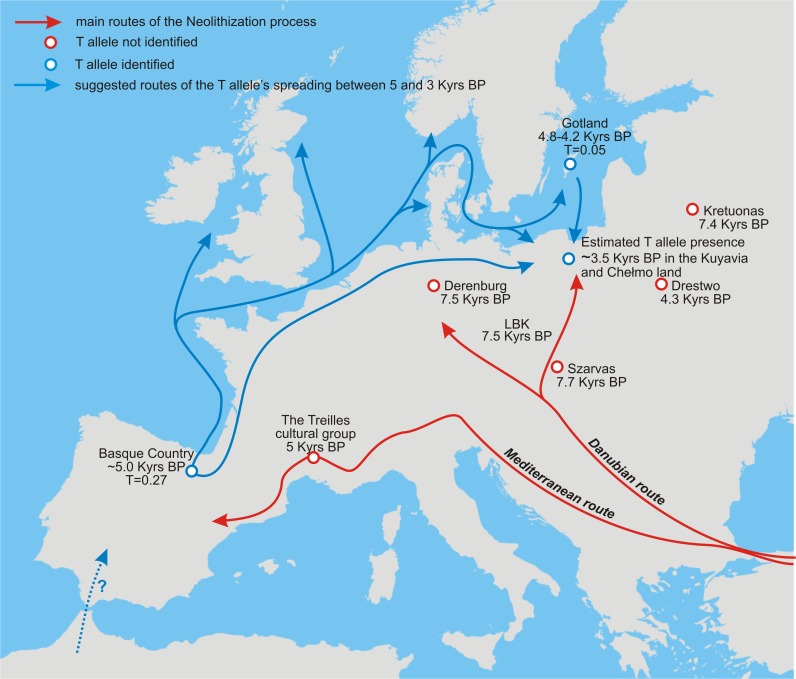
Suggested Northern Route of LCT-13910*T spreading. Contrasted is time-dependent occurrence of the T allele along west-east gradient from Iberia (0.27; >5 Ka BP [66]), through Scandinavia (0.05; >4 Ka BP [67]), up to Kuyavia and the Chełmno land (<4 Ka BP as predicted by us), with its simultaneous absence along the Danubian and Mediterranean Routes.

Leaving aside the currently unsolvable question on the place of origin of the T allele (too scarce data), the observed high frequency in the area of the Basque Country 5.0–4.5 Ka BP [66], in contrast to Central [14] and Southern Europe [13], justifies a speculation on a distinct spreading scenario of the T allele (Fig. 5). It could have spread along a pathway never considered, which we propose to call the Northern Route (NR), running eastward from northern Iberia along European coast by sea and/or by land, reaching regions mostly north of the main stream where high frequency of LCT-13910*T is observed today. Marine NR could have ran along the Biscay Gulf, the Celtic Sea, the English Channel to the North Sea and through Skagerrak and Kattegat to the Baltic Sea, “delivering” the allele not only to the northern part of continental Europe, but also to northwest European archipelagos. It was found that the first farmers arrived in northern Britain, the Channel Isles and the Isle of Man 5.7 Ka BP. They practiced dairying in contrast to the Mesolithic inhabitants of the region and rapidly changed local dietary habits giving up marine resources and adopting intensive dairy farming [68]. Such perfect circumstances would allow for selection of the T allele just after its introduction. Although a different scheme of the Neolithization process was observed in the region of the Baltic Sea, dairying was also practiced since the very beginning of introduction of new technologies [57]. The finding of the T allele in the Mesolithic population from Gotland (PWC), inhabiting the island simultaneously with the Neolithic population of the Funnel Beaker culture (TRB) which practiced dairying since 6.0–5.0 Ka BP [69], suggests that people from Gotland living between 4.8–4.2 Ka BP as well as those from Central Poland (living roughly between 4 and 3 Ka BP) could have witnessed the beginning of the T allele selection, in contrast to individuals from Central and Southern Europe. The Northern Route of the allele’s spreading is even more likely in the light of recently suggested genetic similarity between Scandinavian and Iberian farmers [70].

A kind of temporal and spatial gradient of the T allele frequency found in people from Iberia, Gotland and estimated for Kuyavia might suggest its appearance in time-dependent manner and west-east direction of spreading. However, having such scarce data one can only hypothesize on the T allele spreading from Iberia. The fact that LP in a medieval population from archaeological site in Dalheim, Germany, was 0.72 [71], almost as high as in medieval SBK-4 and Gruczno, might suggest that location along banks of large rivers allowed for enhanced contact with carriers of the T allele, being spread along NR. While such sites as Dalheim were in a direct contact with the southern coast of the North Sea by the Rheine river, the region of Central Poland (SBK-4 and Gruczno) was connected with the Baltic Sea by the Vistula River.

Obviously, verification of the suggested scenario of the T allele spreading route along the Northern Route from Iberia roughly between the 5^th^ and 3^rd^ millennium BP needs many more individuals from over the European archaeological sites to be analyzed for lactase persistence.

### An alternative hypothesis on selection mechanism of alleles involved in lactase persistence

A number of theories to explain LCT-13910*T selection have been proposed, however, none of them was verified [27], even the one regarding calcium and vitamin D supplementation at higher latitudes [72]. We hypothesize that no particular agent or agents were involved in the selection process of the T allele besides the basic living needs related to survival. Although drinking of milk by lactose intolerant adults is not life-threatening, this inability in children after weaning, who face hunger and thirst, could have been. High mortality in such children, which could have resulted mostly from malnutrition and diarrhea together with infections being common even today [73–76], likely favored survival of lactose tolerant children having access to milk available in Neolithic populations [55,77–79]. Besides high mortality, high fertility could become second of driving forces which fueled the T allele selection process in a natural way. Thus, the introduction of the allele into any milking population had to result in the beginning of its frequency changes. Undoubtedly, the disadvantageous and changing environmental conditions affecting crops, as suggested for the Neolithic [60,61,80], which most probably resulted in food shortage, could have been involved in stimulating the selection rate. Thus, the more towards North was an inhabited area located and the harder the living conditions, the stronger the selection pressure operating through altered mortality/fertility rates. Traces of the process were already found in medieval sites in Western (Dalheim site [71]) and Central Europe, as identified by us at SBK-4 and Gruczno sites, but also in Northern Europe today [1,81]. Unfortunately, broader ancient DNA data of the T allele frequency from the British Isles and Scandinavia are not yet available for the comparison.

Also, the type of processes underlying the drop of the T allele’s frequency between the Middle Ages and modern times clearly distinguishes demography of Polish lands from population of more central territory of Europe, e.g. Germany and Austria, where no significant decrease was observed during the last few centuries [71]. A lower average abundance of the T allele observed in moderns living on the Polish territory and comparable to medieval Cedynia and Śródka, as opposed to Kuyavia, implies its rather different history and origin on the area. More detailed studies covering the region over the last 5–7 centuries are needed to explain the observed differences and establish involvement of such agents as migration or pathogens in alteration of the gene pool content.

## Conclusions

1. The presence of the allele/trait in the past should be interpreted very carefully, since we are not able to reconstruct, sometimes even roughly, the agents influencing the profile of changes, as we observed in the case of drastic drop of the T allele/LP between the studied medieval populations, as well as between medieval and modern times. The best approach to establish mechanisms driving such processes in the past seems to be achievable through typing more numerous samples which are differentiated temporally and spatially.
2. One can speculate that in milk-producing and dairy farming populations, both high mortality and fertility could have been involved in shaping the rate of LP alleles’ selection process, resulting in higher survival rate of lactase persistent post-weaning children, an effect pronounced under challenging living conditions. Such mechanism might modulate the selection process of lactase persistence alleles both in cold Northern Europe as well as warm and arid regions of north-western Africa and the Arabian Peninsula. In fact, agents influencing mortality in post-weaning children *via* restricted access to food and water in population practicing cattle breeding and milk processing should be considered as presumably involved in the selection of LP alleles.
3. The Northern Route of LCT-13910*T spreading seems to be very likely, however, data from more samples and sites are needed to verify the hypothesis.

The mtDNA sequences discussed in the paper and presented in supplementary data (Tables D-K in S1 File) can be found in the NCBI GenBank (http://www.ncbi.nlm.nih.gov/genbank/) under accession numbers KM986326—KM986456.

## Supporting Information

S1 Fig The assumed population size over generations. (TIF)

S2 Fig Comparison of LP frequency as calculated from the T allele frequency (green dots) and from random drawing of genotypes from the allele pool (blue dots), together with difference between the two data sets (red dots); the allele’s introduction time about 315 generations BP, selection coefficient 0.03. (TIF)

S1 File Content of S1 File: Table A. Yield of DNA isolation at the studied archaeological sites. **Table B.** Dating of the studied samples. **Table C.** mtDNA haplotypes and LCT-13910 alleles in people involved in processing of the studied skeletal material. **Table D.** mtDNA haplotype and the LCT-13910 allele in individuals from the Neolithic. GAC—Globular Amphora culture, LC—Lengyel culture. **Table E.** mtDNA haplotype and the LCT-13910 allele in Hallstatt people from Gzin, Pędzewo and Grodno. **Table F.** mtDNA haplotype and the LCT-13910 allele in people from Linowo. **Table G.** mtDNA haplotype and LCT-13910 allele in people from Rogowo. **Table H.** mtDNA haplotype and the LCT-13910 allele in people from Gruczno. **Table I.** mtDNA haplotype and the LCT-13910 allele in people from SBK-4. **Table J.** mtDNA haplotype and the LCT-13910 allele in people from Cedynia. **Table K.** mtDNA haplotype and the LCT-13910 allele in people from Śródka. **Table L.** Key dates considered during calculations of time of the T allele introduction and beginning of lactase persistence selection in the region of Kuyavia and the Chełmno land, Poland.
(DOCX)
