# Supplementary figures and images for: Hunting for the LCT-13910*T Allele between the Middle Neolithic and the Middle Ages Suggests Its Absence in Dairying LBK People Entering the Kuyavia Region in the 8th Millennium BP
Source: PLoS One. 2015 Apr 8;10(4):e0122384. doi: 10.1371/journal.pone.0122384 (PMC4390234; doi:10.1371/journal.pone.0122384)

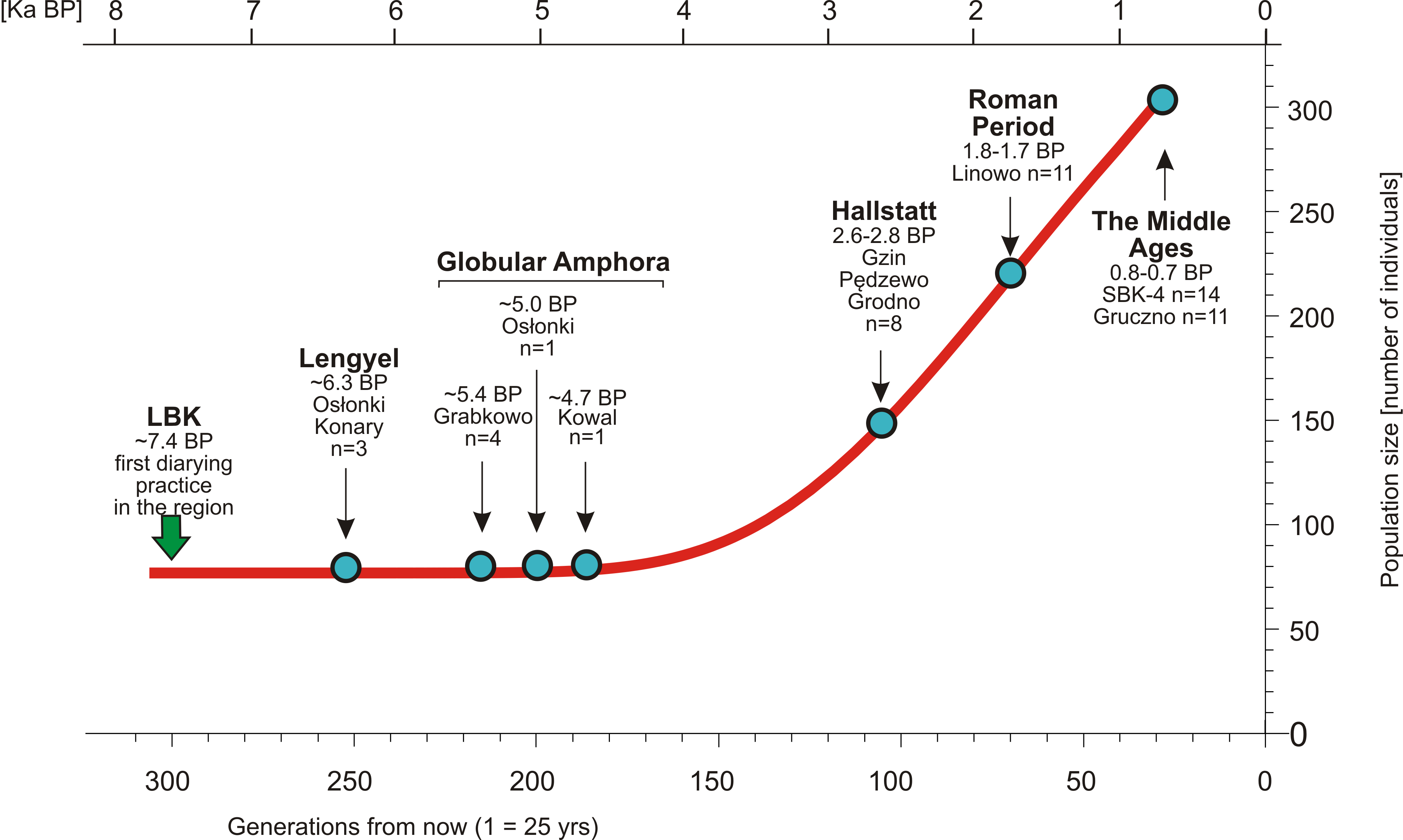

Supplement: S1 Fig — (TIF) [file pone.0122384.s001.tif]

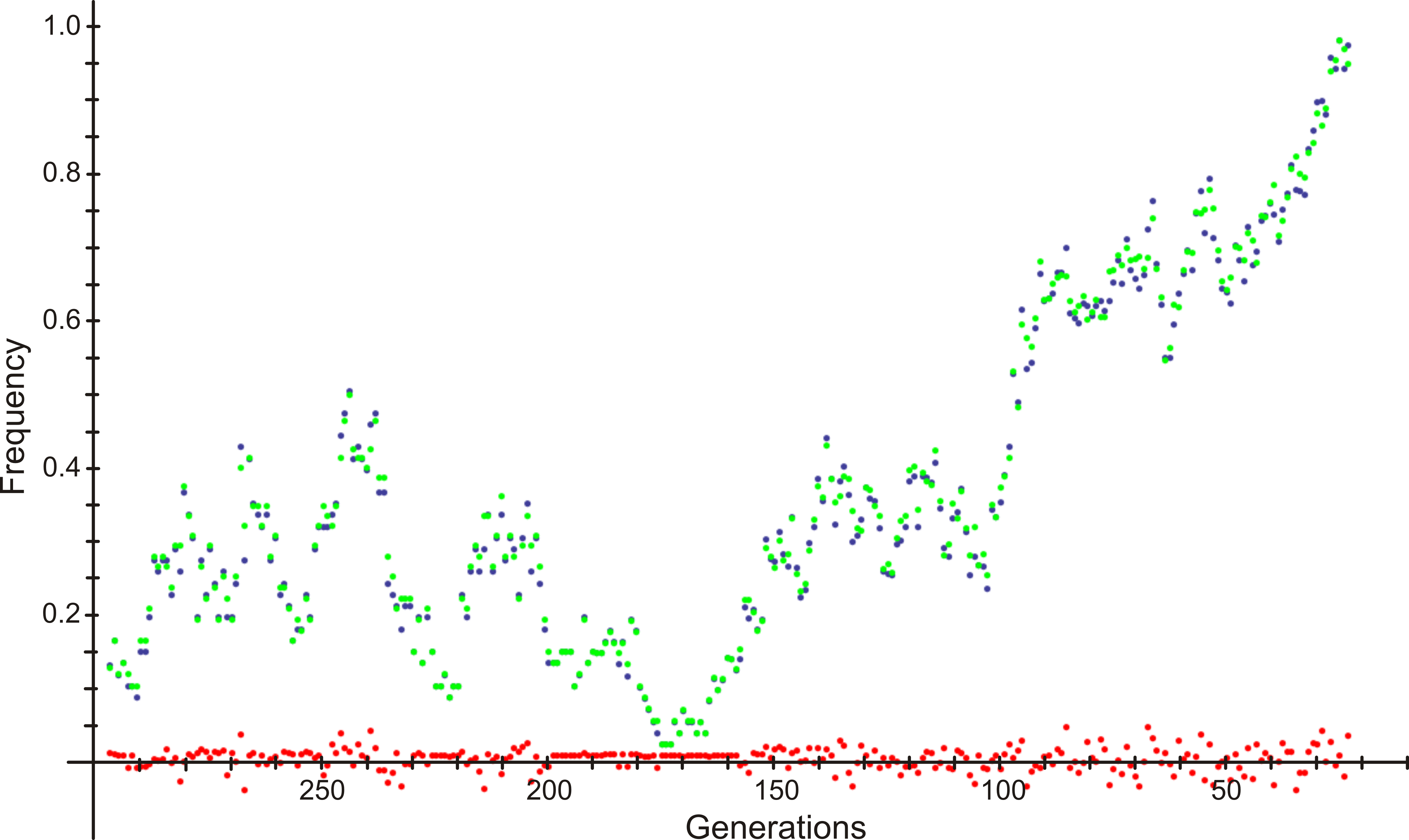

Supplement: S2 Fig — (TIF) [file pone.0122384.s002.tif]
