# Supplementary material for: Hunting for the LCT-13910*T Allele between the Middle Neolithic and the Middle Ages Suggests Its Absence in Dairying LBK People Entering the Kuyavia Region in the 8th Millennium BP
Source: PLoS One. 2015 Apr 8;10(4):e0122384. doi: 10.1371/journal.pone.0122384 (PMC4390234; doi:10.1371/journal.pone.0122384)
Supplement: S1 File — Table B. Dating of the studied samples. Table C. mtDNA haplotypes and LCT-13910 alleles in people involved in processing of the studied skeletal material. Table D. mtDNA haplotype and the LCT-13910 allele in individuals from the Neolithic. GAC—Globular Amphora culture, LC—Lengyel culture. Table E. mtDNA haplotype and the LCT-13910 allele in Hallstatt people from Gzin, Pędzewo and Grodno. Table F. mtDNA haplotype and the LCT-13910 allele in people from Linowo. Table G. mtDNA haplotype and LCT-13910 allele in people from Rogowo. Table H. mtDNA haplotype and the LCT-13910 allele in people from Gruczno. Table I. mtDNA haplotype and the LCT-13910 allele in people from SBK-4. Table J. mtDNA haplotype and the LCT-13910 allele in people from Cedynia. Table K. mtDNA haplotype and the LCT-13910 allele in people from Śródka. Table L. Key dates considered during calculations of time of the T allele introduction and beginning of lactase persistence selection in the region of Kuyavia and the Chełmno land, Poland. (DOCX) [file pone.0122384.s003.docx]

SUPPORTING INFORMATION – File S1

Table A. Yield of DNA isolation at the studied archaeological sites.

| **Site/culture** | **Number of studied individuals (n)** | **% yield  (n)** |
| --- | --- | --- |
| Cedynia | 47 | 74.5 (35) |
| Śródka | 20 | 80.0 (16) |
| SBK 4 | 46 | 30.4 (14) |
| Gruczno | 30 | 50.0 (15) |
| Rogowo | 21 | 100.0 (21) |
| Linowo | 18 | 72.2 (13) |
| The Hallstatt | 17 | 47.0 (8) |
| The Neolithic | 32 | 28.1 (9) |
|  | **231** | **60.3 (131)** |

Table B. Dating of the studied samples.

| **Archaeological site** | **Dates [Ka BP]** | **Dating method** | **Archaeological culture** |
| --- | --- | --- | --- |
| Cedynia | 1.0-0.8 | grave equipment | medieval |
| Sródka | 1.0-0.8 | grave equipment | medieval |
| SBK-4 | 0.6-0.6 | grave equipment | medieval |
| Gruczno | 0.6-0.6 | grave equipment | medieval |
| Linowo, Rogowo | 1.8-1.7 | grave equipment | medieval |
| Gzin, Pędzewo, Grodno | 2.8-2.6 | dendrochronological | Hallstatt |
| Kowal | 4.87-4.5 | C^14^ | Globular Amphora |
| Grabkowo | 5.5-5.36 | C^14^ | Globular Amphora |
| Osłonki | 5.1-4.9 | C^14^ | Globular Amphora |
| Osłonki | 6.5-6.3 | C^14^ | Lengyel |
| Osłonki | 6.3-6.1 | C^14^ | Lengyel |
| Konary | 6.4-6.2 | C^14^ | Lengyel |

Table C. mtDNA haplotypes and LCT-13910 alleles in people involved in processing of the studied skeletal material.

|  | mtDNA haplotype | LCT-13910 C/T |
| --- | --- | --- |
| TP | 16223T 16297C 16298C 16327T | C/C |
| KJ | 16189C 16270T 16291T | C/T |
| BJ | 16129A 16172C 16223T 16311C | T/T |
| GO | 16288C | C/C |
| WL | 16192T | C/C |
| PP | 16146G 16294T | C/T |
| TK | 16126C | C/T |
| EŻ | 16189C | T/T |
| AM | 16224C 16284G 16311C 16319A | C/T |
| HW | CRS | C/T |

Table D. mtDNA haplotype and the LCT-13910 allele in individuals from the Neolithic. GAC – Globular Amphora culture, LC – Lengyel culture.

|  |  | **Culture** | **mtDNA haplotype** | **macro-hg** | **hg** | **LCT-13910** |
| --- | --- | --- | --- | --- | --- | --- |
| 1 | Kowal | GAC | 16224C 16311C | K | K | CC |
| 2 | PN11 | GAC | CRS | H | H | CC |
| 3 | K7 | GAC | 16224C 16311C | K | K | CC |
| 4 | K22 | GAC | 16189C | H | H1 | CC |
| 5 | K21 | GAC | 16311C | H | H2b | CC |
| 6 | L1 | GAC | 16126C 16193T | J | J1d | CC |
| 7 | L3 | LC | CRS | H | H | CC |
| 8 | L6 | LC | CRS | H | H | CC |
| 9 | L7 | LC | 16126C 16294T 16296T 16304C | T | T2b | CC |

Table E. mtDNA haplotype and the LCT-13910 allele in Hallstatt people from Gzin, Pędzewo and Grodno.

|  |  | **mtDNA haplotype** | **macro-hg** | **hg** | **LCT-13910** |
| --- | --- | --- | --- | --- | --- |
| 1 | K62 | CRS | H | H | C/C |
| 2 | K65 | 16184T | H | H15a1b | T/T |
| 3 | K66 | 16126C | H | H | C/C |
| 4 | K44 | 16189C | H | H1 | C/T |
| 5 | K46 | 16189C 16270T | U5 | U5b1b1 | C/C |
| 6 | K47 | 16172C 16222T 16261T | H | H | C/C |
| 7 | K49 | 16224C 16311C | K | K | C/C |
| 8 | K50 | 16294T 16296T 16304C | T | T2b | C/C |

Table F. mtDNA haplotype and the LCT-13910 allele in people from Linowo.

|  |  | **mtDNA haplotype** | **macro-hg** | **hg** | **LCT-13910** |
| --- | --- | --- | --- | --- | --- |
| 1 | K91 | 16294T 16296T 16304C | T | T2b | CT |
| 2 | K92 | 16162G | H | H1a | CC |
| 3 | K93 | 16169T 16299G | H | H39a | CC |
| 4 | K96 | 16126C 16163G 16186T 16189C 16294T | T | T1a | CC |
| 5 | K97 | 16294T 16296T 16304C | T | T2b | CT |
| 6 | K90 | CRS | H | H | - |
| 7 | K98 | 16162G 16291T 16304C | H | H1a | CC |
| 8 | K100 | CRS | H | H | TT |
| 9 | K101 | 16129A 16148T 16223T 16269C | I | I5a | CT |
| 10 | K102 | 16126C | H | H | CT |
| 11 | K103 | 16126C 16294T 16304C | T | T2b | CT |
| 12 | K104 | 16140C 16293G 16311C | H | H11a2a | - |
| 13 | K106 | 16145A 16189C 16198T 16256T 16270T | U5 | U5a1d2a | CT |

Table G. mtDNA haplotype and LCT-13910 allele in people from Rogowo.

|  |  | **mtDNA haplotype** | **macro-hg** | **hg** | **LCT-13910** |
| --- | --- | --- | --- | --- | --- |
| 1 | K52 | 16316G | H | H27 | CC |
| 2 | K53 | 16189C | H | H1 | CC |
| 3 | K54 | 16224C 16311C | K | K | TT |
| 4 | K55 | 16304C | H | H5 | TT |
| 5 | K56 | CRS | H | H | CT |
| 6 | K57 | 16304C | H | H5 | TT |
| 7 | K58 | 16126C | H | H | CT |
| 8 | K59 | CRS | H | H | CC |
| 9 | K38 | 16189C | H | H1 | CT |
| 10 | K39 | 16209C | H | H5a1j or H1 | CT |
| 11 | K40 | CRS | H | H | CC |
| 12 | K42 | 16304C | H | H5 | CT |
| 13 | K43 | 16304C | H | H5 | TT |
| 14 | K67 | 16298C | HV | HV0 | - |
| 15 | K68 | 16126C | H | H | CC |
| 16 | K69 | 16189C | H | H1 | CC |
| 17 | K70 | CRS | H | H | CT |
| 18 | K71 | CRS | H | H | TT |
| 19 | K72 | CRS | H | H | TT |
| 20 | K73 | 16189C 16272G | H | H10g | CT |
| 21 | K88 | 16304C | H | H5 | TT |

Table H. mtDNA haplotype and the LCT-13910 allele in people from Gruczno.

|  |  | **mtDNA haplotype** | **macro-hg** | **hg** | **LCT-13910** |
| --- | --- | --- | --- | --- | --- |
| 1 | K15 | 16224C 16311C | K | K | CT |
| 2 | K25 | 16294T 16304C | H | H5a4 | CT |
| 3 | K30 | 16294T 16296T 16324C | T | T2a1b | CC |
| 4 | K31 | 16311C | H | H2a | TT |
| 5 | K74 | 16172C 16222T 16294T 16296T | T | T2 | TT |
| 6 | K75 | 16134T 16172C | H | H | TT |
| 7 | K76 | 16192T 16256T 16270T 16294T | U | U5a2a | CT |
| 8 | K32 | 16179T | H | H | - |
| 9 | K33 | 16125T 16234T | U | U2c | - |
| 10 | K77 | CRS | H | H | - |
| 11 | K78 | 16309C | H | H | CC |
| 12 | K108 | 16288C 16311C | H | H8 | TT |
| 13 | K109 | CRS | H | H | - |
| 14 | K110 | CRS | H | H | TT |
| 15 | K111 | 16298C | HV | HV0 | CT |

Table I. mtDNA haplotype and the LCT-13910 allele in people from SBK-4.

|  |  | **mtDNA haplotype** | **macro-hg** | **hg** | **LCT-13910** |
| --- | --- | --- | --- | --- | --- |
| 1 | SBK 159 | 16189C | H | H1 | CT |
| 2 | SBK 166 | 16187T 16234T | H | H | CT |
| 3 | SBK 167 | 16256T 16293G | H | H24 | CT |
| 4 | SBK 168 | 16234T | H | H | CC |
| 5 | SBK 170 | 16265G | H | H7c1 | CT |
| 6 | SBK 171 | CRS | H | H | CT |
| 7 | SBK 172 | 16234T | H | H | CT |
| 8 | SBK 177 | CRS | H | H | CC |
| 9 | SBK 180 | CRS | H | H | CT |
| 10 | SBK 165 | 16234T 16270T 16272G | U5 | U5b1d | TT |
| 11 | SBK 169 | 16239T 16256T 16270T 16311C | U5 | U5a | TT |
| 12 | SBK 174 | 16192T 16248T 16256T 16270T | U5 | U5a | CT |
| 13 | SBK 161 | 16224C 16270T 16311C | K | K | CT |
| 14 | SBK 157 | 16298C | HV | HV0 | TT |

Table J. mtDNA haplotype and the LCT-13910 allele in people from Cedynia.

|  |  | **mtDNA haplotype** | **macro-hg** | **hg** | **LCT-13910** |
| --- | --- | --- | --- | --- | --- |
| 1 | B809 | 16148T | H | H | CC |
| 2 | B470 | 16104T 16286T 16311C | H | H2b | CC |
| 3 | B121 | 16316G | H | H7 | CT |
| 4 | B404 | CRS | H | H | CC |
| 5 | B447 | CRS | H | H | CC |
| 6 | B475 | 16140C 16265G 16311C 16327T | H | H7c1 | CC |
| 7 | B546 | 16126C 16304C | H | H | CT |
| 8 | B944 | CRS | H | H | CC |
| 9 | B1072 | CRS | H | H | CT |
| 10 | B1186 | CRS | H | H | CC |
| 11 | B208 | CRS | H | H | CC |
| 12 | B53 | 16288C 16290T | H | H8 | CT |
| 13 | B621 | 16327T | H | H | CT |
| 14 | B759 | CRS | H | H | CC |
| 15 | B794 | 16292T | H | H | CC |
| 16 | B941 | 16189C | H | H1 | CT |
| 17 | B67 | CRS | H | H | CT |
| 18 | B14 | 16224C 16311C | K | K | CC |
| 19 | B423 | 16224C 16311C | K | K | CT |
| 20 | B799 | 16224C 16311C | K | K | CC |
| 21 | B549 | 16304C | H | H5 | CT |
| 22 | B1159 | 16294T 16296T 16304C | T | T2b | CC |
| 23 | B1105 | 16270T 16311C | U5 | U5b1d | CT |
| 24 | B100 | 16192T 16256T 16270T | U5 | U5a | CT |
| 25 | B 402 | 16298C | HV | HV0 | CT |
| 26 | B492 | 16192T 16270T 16274A | U5 | U5 | CC |
| 27 | B1201 | 16294T 16296T | T | T2 | CC |
| 28 | B1205A | 16287C 16294T 16296T | T | T2 | CT |
| 29 | B1186 | CRS | H | H | CC |
| 30 | B429 | 16192T 16270T | U5 | U5 | CC |
| 31 | B553 | 16249C 16256T | H | H | CC |
| 32 | B739 | 16270T | U5 | U5 | CC |
| 33 | B956 | CRS | H | H | CT |
| 34 | B128 | 16185T 16223T 16224C 16260T | Z | Z1a | CC |
| 35 | B490 | 16189C 16234T 16260T 16287T 16294T | U2 | U2d | CC |

Table K. mtDNA haplotype and the LCT-13910 allele in people from Śródka.

|  |  | **mtDNA haplotype** | **macro-hg** | **hg** | **LCT-13910** |
| --- | --- | --- | --- | --- | --- |
| 1 | P1 | 16256T 16270T 16294T 16295T | U5 | U5a | CC |
| 2 | P3 | 16270T | U5 | U5 | CC |
| 3 | P7 | 16223C | H | H | CT |
| 4 | P10 | CRS | H | H | CC |
| 5 | P9 | 16168T | H | H7f | CT |
| 6 | P11 | 16304C | H | H5 | CC |
| 7 | P12 | 16266T | H | H1t1 | CC |
| 8 | P14 | 16294T 16304C | H | H5a4 | CT |
| 9 | P2 | 16298C | HV | HV0 | - |
| 10 | P13 | CRS | H | H | CC |
| 11 | P8 | 16145A 16172C 16222T 16261T | J | J1b1a1 | - |
| 12 | P15 | 16189C | H | H1 | TT |
| 13 | P16 | 16192T 16270T | U5 | U5 | CC |
| 14 | P17 | 16192T 16256T 16270T 16294T | U5 | U5a | CT |
| 15 | P19 | 16145A 16172C 16222T 16261T | J | J1b1a1 | CC |
| 16 | P20 | CRS | H | H | TT |

Table L. Key dates considered during calculations of time of the T allele introduction and beginning of lactase persistence selection in the region of Kuyavia and the Chełmno land, Poland.

| **Culture/Period** | **Site** | **Date [Ka BP]** | **T allele** | **LP** |
| --- | --- | --- | --- | --- |
| Neolithic | Osłonki | 6.3 | - | - |
|  | Grabkowo | 5.425 | - | - |
|  | Kowal | 4.675 | - | - |
| Hallstatt | | 2.7 | 0.19 | 0.25 |
| Roman | | 1.75 | 0.36 | 0.64 |
| Middle Ages | | 0.7 | 0.56 | 0.84 |
